# Supplementary material for: Neurodegeneration risk factor, EIF2AK3 (PERK), influences tau protein aggregation
Source: J Biol Chem. 2022 Dec 21;299(2):102821. doi: 10.1016/j.jbc.2022.102821 (PMC9852698; doi:10.1016/j.jbc.2022.102821)
Supplement: Supplementary figures [file mmc1.pdf]

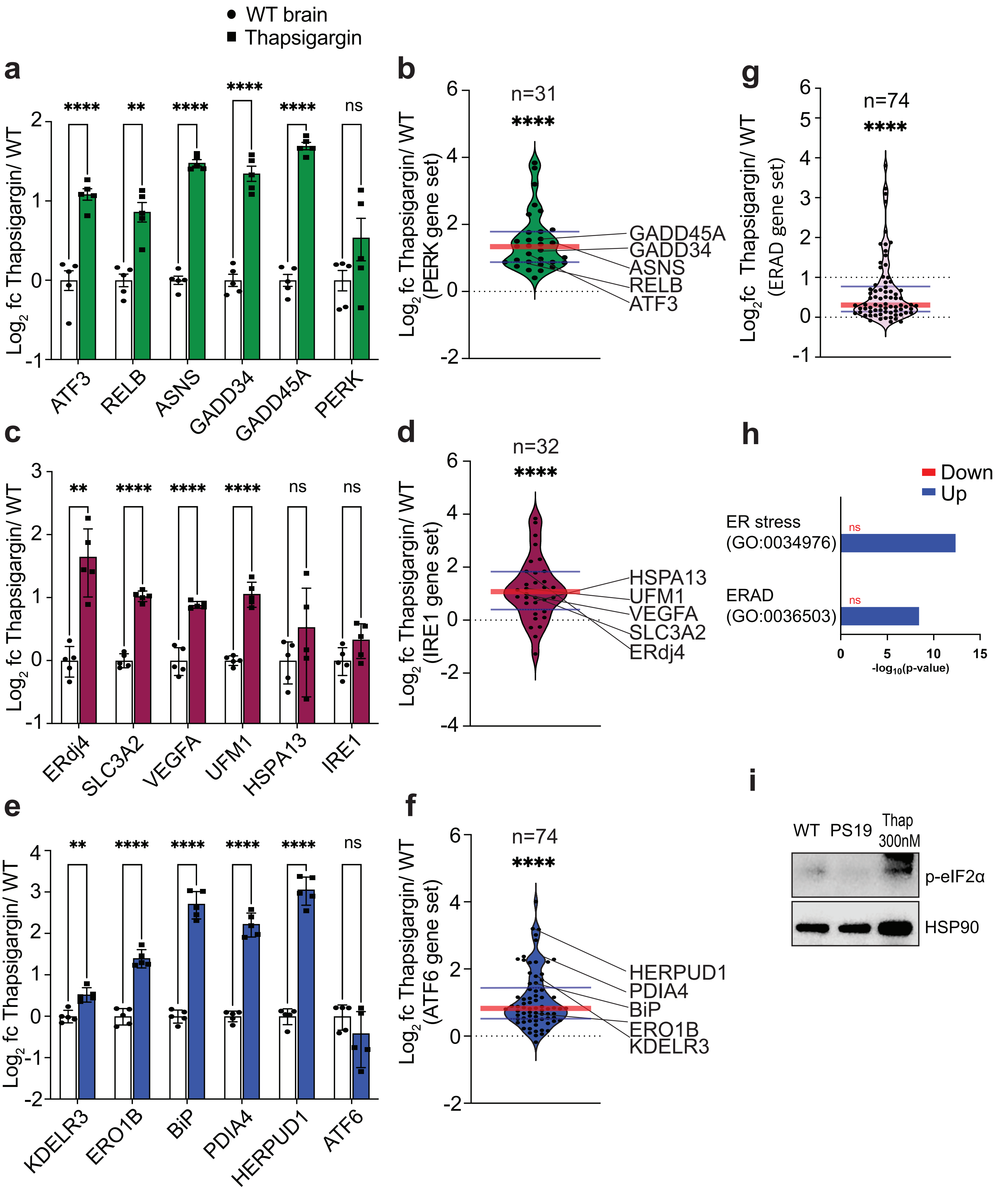

Suppl. Figure 1

a

NES: -0.82
NOM p-val: 0.938
FDR q-val: 1.000

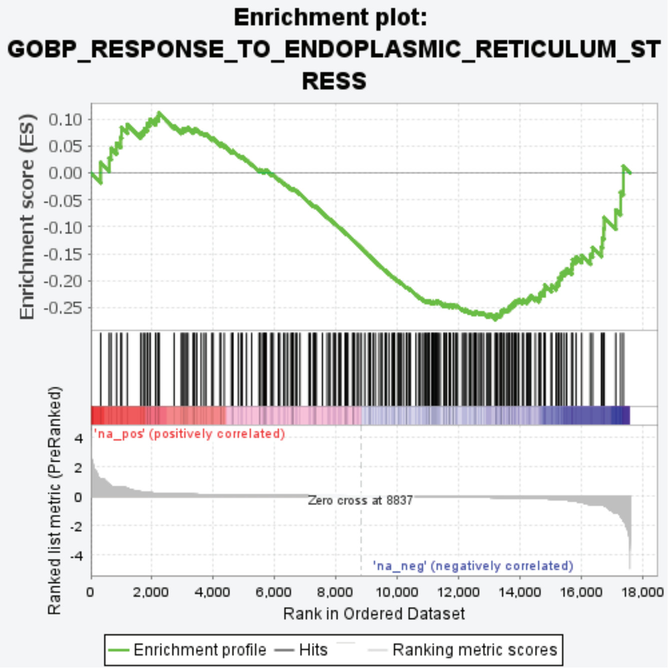

b

NES: 0.46
NOM p-val: 0.992
FDR q-val: 1.000

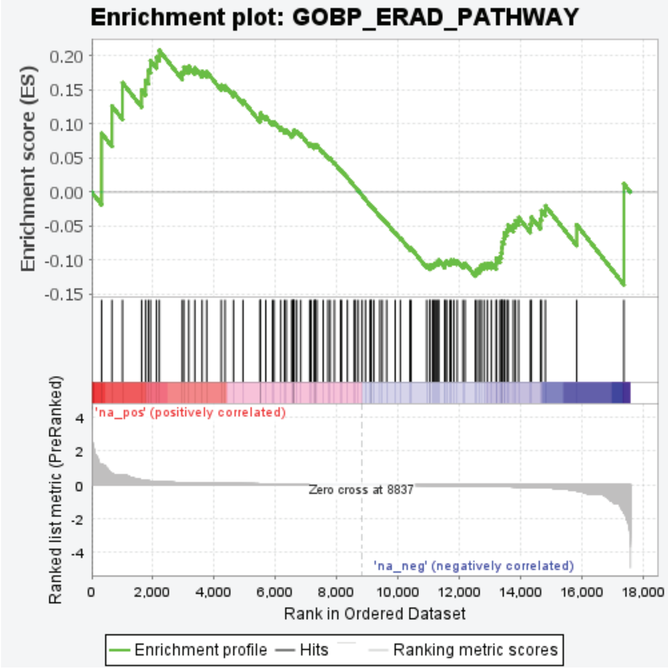

c

NES: 2.32
NOM p-val: 0.000
FDR q-val: 0.000

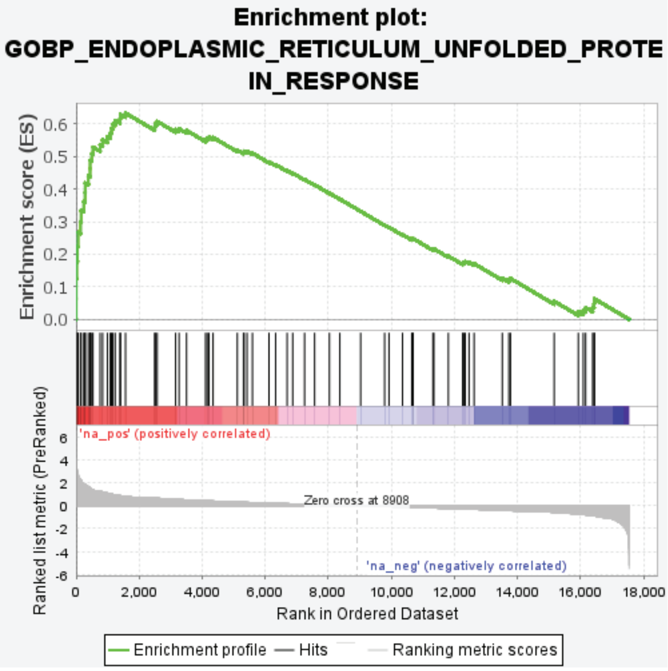

d

NES: 1.89
NOM p-val: 0.000
FDR q-val: 0.042

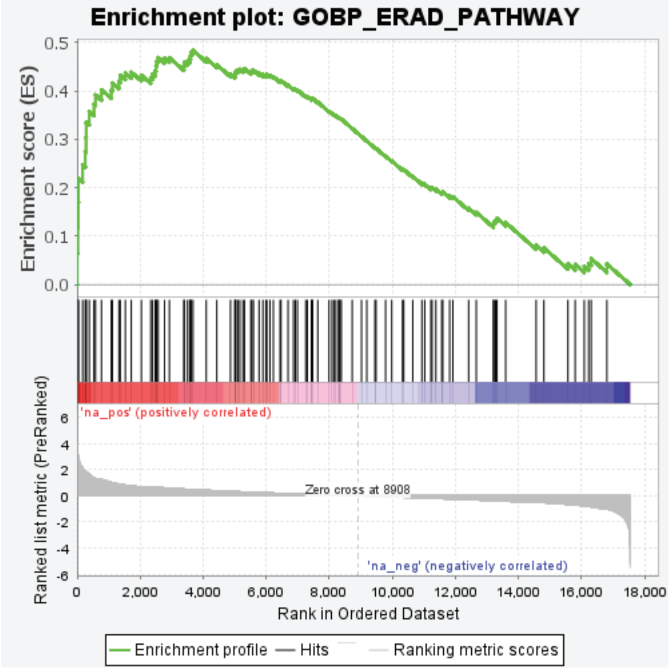

Suppl. Figure 2

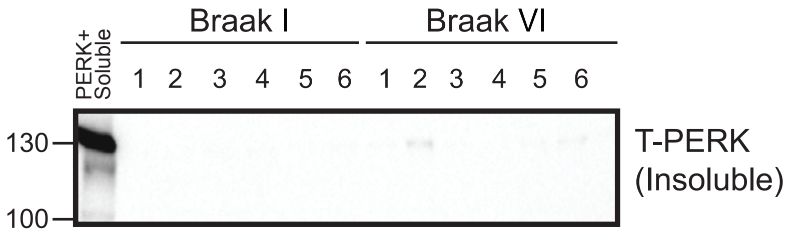

Suppl. Figure 3
